# Automatic system for the determination of boron in ceramic frits

**DOI:** 10.1155/S1463924691000202

**Published:** 1991

**Authors:** F. Más, J. M. Estela, V. Cerdà, E. Ochandio

**Affiliations:** 1 Department of Chemistry University of the Balearic Islands Palma de Mallorca 07071 Spain; 2 Asociación Española de Industrias Cerámicas (AICE), Paterna Valencia Spain; 3 Instituto Technológico Cerámico (ITC) Universidad de Valencia Spain

## Abstract

An automatic system for the potentiometric determination of boron in ceramic frits was developed. The system includes a personal computer for instrumental control, data acquisition and processing, which allows up to 13 samples to be analysed sequentially with no human intervention.

The system performance was tested on the titration of standard solutions, which it carried out with low errors and RSD. It was subsequently applied to the determination of the B_2_0_3_ content in various types of ceramic frits with good results.


Journal of Automatic Chemistry, Vol. 13, No. 3 (May–June 1991), pp. 107–110

### Automatic system for the determination of boron in ceramic frits

**F. Más, J. M. Estela, V. Cerdà**

*Department of Chemistry, University of the Balearic Islands, 07071 Palma de Mallorca, Spain*

**and E. Ochandio**

*Asociación Española de Industrias Cerámicas (AICE), Paterna, Valencia, Spain*

*Instituto Technológico Cerámico (ITC), Universidad de Valencia, Spain*

*An automatic system for the potentiometric determination of boron in ceramic frits was developed. The system includes a personal computer for instrumental control, data acquisition and processing, which allows up to 13 samples to be analysed sequentially with no human intervention.*

*The system performance was tested on the titration of standard solutions, which it carried out with low errors and RSD. It was subsequently applied to the determination of the $B_2O_3$ content in various types of ceramic frits with good results.*

### Introduction

Boron, as boric oxide ($B_2O_3$), is an important chemical component of many raw materials and a variety of frits used as intermediate products by the ceramic industry [1–3]. The Asociación Espanola de Industrias Cerámicas (AICE) laboratories have been analysing this type of ceramic material by X-ray fluorescence (XRF) for five years [4]. However, the equipment currently available (Philips PW 1410) does not allow boron to be assayed, so manual potentiometric titrations have to be performed to quantify this element [5].

The advent of personal computers and scientific instrumentation that can be controlled by them have been significant steps towards the automation of research and routine laboratory processes. In earlier work [6–12] the advantages of these systems over instrumentation based on the use of dedicated microprocessors have been described: one of the most significant being the possibility of programming in affordable high-level languages, rather than the unmodifiable programs developed by manufacturers in low-level languages and stored in read-only memory (ROM). This allows automation to be addressed in a modular fashion as regards both hardware and software. With a stock of a few basic instrumental modules it is possible to assemble inexpensive automatic instrumental systems by simply altering the block diagram of the starting set-up.

Using modular software routines it is extremely easy to develop new programs for specific instrumental systems as this paper demonstrates. The example reported here involved adapting a potentiometric system used for general routine research to performing automatic potentiometric titrations of boron in ceramic materials. The significance of this determination lies in the fact that the chemical composition of each frit determines some of its features and properties (for example melting point, chemical and mechanical resistance, surface, tension). The boron oxide content of these products ranges between 5 and 10% in matt and opaque, 5 and 15% in transparent, 0 and 20% in lead, and 20% and 40% in boric frits.

### Experimental

### *Apparatus*

The instrumental set-up used consisted of the following components:

(1) A PC with two RS232C serial ports and a printer.

(2) A Crison Instruments SA (Riera Principal 24–26, 08328 Allela, Barcelona, Spain) 2002 potentiometer with an RS232C communication interface.

(3) Two Crison microBur 2031 autoburettes, also with RS232C interfaces.

(4) A Crison microSampler 2040 autosampler with an RS232C interface and a mechanical stirrer.

(5) An Ingold combined glass-Ag/AgCl pH electrode.

### *Reagents*

The solutions used included 0·0096 M NaOH titrated with potassium hydrogen phthalate, 0·06 M HCL, 0·1 M boric acid and 7·5% mannitol. All reagents used were Merck r.a. grade; distilled water was used throughout.

### *Procedure for the automatic potentiometric determination of boron in frits*

#### *Sample disaggregation*

Samples are solubilized by alkaline fusion with an equimolar mixture of sodium and potassium carbonate. The melt is dissolved in HCl, neutralized with solid calcium carbonate and refluxed for 10 min. It is then filtered through paper of medium porosity, acidified with a few drops of HCl and refluxed for 30 min [1].

#### *Automatic potentiometric titration*

The pH electrode is calibrated with buffers of pH 4 and 7. The disaggregated samples, or selected aliquots, are placed in the cups of the autosampler, which allows up to 13 samples to be titrated sequentially – two of the 15 cups are devoted to cleaning the electrode between samples. After starting the program (BORIC), samples are identified and other data requested by the program are entered as requested through screen menus. Then, the program commands and controls the analysis of the 13 samples and lists the boron content of each sample.

0142–0453/91 $3.00 © 1991 Taylor & Francis Ltd.

The automatic titration procedure includes the following steps: first, the excess HCl used to neutralize the sodium carbonate employed in the disaggregation step is titrated with NaOH from the first autoburette. Then, mannitol is added from the second autoburette and the titration with NaOH is resumed to measure the boric acid originally present in the sample. The titrant addition is controlled automatically by an algorithm that takes account of the potential increment per added titrant volume.

### *Hardware*

Figure 1 shows the scheme of the set-up used for the automatic titration of boric acid from the disaggregation of ceramic frits. Note that the specifications of the different modules supplied by the manufacturers necessitate the use of two RS232C communication boards on a PC computer. The potentiometer operates at a rate of 1200 baud, while the burettes and the sampler both work at 2400 baud and can be connected serially along the same communication line. The autosampler should be placed last in the serial communication chain.

### *Software*

In developing the proposed automatic potentiometric titration for boron the following points were taken into account:

(1) Establishment of criteria for stabilization of pH readings.

(2) Criteria for caculation of titrant additions according to the titration zone. These were aimed at minimizing the time required to titrate each sample.

(3) Automatic identification of the two equivalence points in order to accurately calculate the boric acid content of each sample.

Earlier studies on routine acid-base potentiometric titrations revealed that satisfactory results can be obtained without waiting for the complete stabilization of the pH or mV reading [7]. The criterion used in this work was waiting for 10 s after each titrant addition before the pH was measured.

The program starts with a menu offering the user the following options: (*a*) titration; (*b*) reading disk; and (*c*) end of the session. This last option returns control to the system and closes down the communication channels.

#### *Option 1: Titration*

This option enables automatic implementation of the titration and control of various peripherals, and includes the following steps:

(1) Dimensionalizing the variables used to keep data.

(2) Naming the file in which the titration conditions and some parameters identifying the method (for example, titrant, sample, sample volume, electrode cleansing cycles, number of samples, volume of the autoburette syringe containing the titrant, overall sample volume) will be stored.

These parameters can be altered and/or stored in the same or in different files. The names of the titrant and sample are identifying parameters. The sample volume is referred to the aliquot taken from the solution obtained on disaggregation of the frit (overall sample volume). Volumes larger than 50 ml are inadvisable because the capacity of the sampler cups is about 75 ml.

One or two electrode cleansing cycles can be implemented, so the maximum number of samples that can be assayed is reduced to 13 or 11, respectively.

Insofar as the autoburette syringe is exchangeable, the volume containing the titrant is required to calculate the number of motor steps needed to shift the autoburette piston in each addition according to:

Number of steps = volume to be added/(syringe volume $\times$2500), where 2500 is the overall number of motor steps corresponding to the difference between the lowest and highest position of the piston in the burette. This parameter also allows the user to know the status of the burette at any time. The volume of the burette containing the mannitol is set by the program because this reagent is added to enhance the acidity of boric acid; however, it can be altered by the user as required.

The overall sample volume coincides with that of the solution resulting from the disaggregation process as long as all samples are subjected to the same treatment.

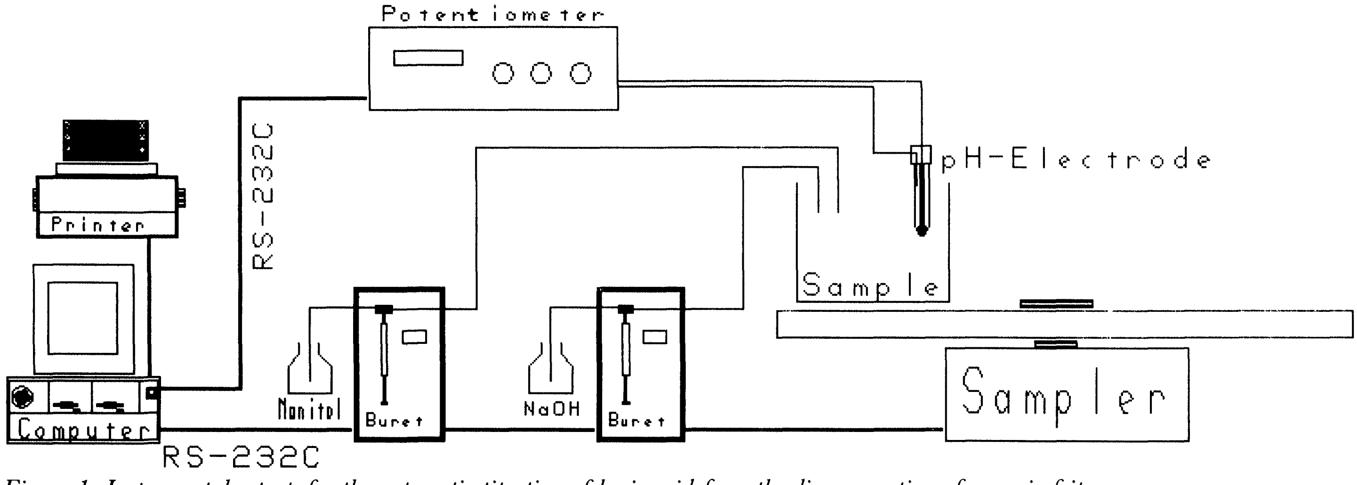

*Figure 1. Instrumental set-up for the automatic titration of boric acid from the disaggregation of ceramic frits.*

All of the above parameters are common to a series of samples subjected to the same procedure; other conditions, such as the time lapse between titrant additions and readings (10 s) and the overall solution volume a cup can contain (75 ml), are set by the program and cannot be altered by the operator.

Once all conditions have been established, the program requests the user to enter each sample name and weight. This latter information is used to calculate the boric oxide content according to:

$$\%B_2O_3 = 3{\cdot}48 V_{eq} C_t f / w_s$$

where $V_{eq}$ denotes the equivalence volume, $C_t$ the molar concentration of the titrant, f a dilution factor equal to $V_{sample}/V_{aliquot}$ and $w_s$ the weight of the disaggregated sample.

These data can be printed out in addition to a header including the file name, date, name of the titrant, overall sample volume and titrated sample volume.

The next step in the process involves opening the two communication channels (pH meter and burettes-sampler), checking the identifying response of each module and opening the file to store the results. Next, the experimental procedure is started by cleansing the electrode, placing the first sample to be titrated under the autoburette and feeding the two burettes prior to starting the titration proper. This sequence is repeated after each titration, so it is started with full burettes.

During the titration, the current added volume and pH are continuously displayed in the upper right corner of the computer screen; the rest of the display is filled with the graph of pH against titrant volume (in ml).

Each new titrant volume to be added is reset according to the pH reading obtained. Also, the program determines whether the titrant burette has been refilled, and, if the cumulative volume exceeds 75 ml – the maximum capacity of the sampler cup, it prompts the user to dilute the sample and titrate the next in the series.

The titration is controlled and equivalence points are determined according to the following criteria:

(*a*) The titration is finished if the pH exceeds 10·5.

(*b*) If the starting pH is lower than 6, then VOL1 = 0 (i.e. HCl is considered not to be in excess).

(*c*) If it is a maximum (dpH/dmV) and pH > 6, then the end-point corresponds to VOL1 or VOL2 (the HCl and boric acid volume, respectively).

(*d*) After each mannitol addition, if the pH is above 8, and if this is the last point in the determination of HCl, then there is no boric acid and hence VOL2 = VOL1, so $V_{eq}$ = VOL2 − VOL1.

The titrant volume increment depends on increases with the pH of the titrand. The criteria used differ according to whether HCl or boric acid is being titrated – the titration of the latter gives rise to less marked pH changes.

If a print-out has been requested, then the sample number and weight, equivalence volume and $B_2O_3$ contents after each titration are given. This information is also displayed on the screen, which clears when the titration process is resumed. This is continued until the present number of samples have been titrated, after which the results are summarized on the screen as a table listing the same data delivered in the printed report.

*Option 2: Disk reading*

This allows the results obtained with the previous option to be surveyed and has two alternatives: either the graph of a given titration can be displayed (option G), or the results for a series of samples (option T). A list with available files for the chosen option is displayed. The user

*Table 1. Results and statistics obtained in the titration of different volumes of boric acid with sodium hydroxide in the presence of hydrochloric acid.*

| $H_3BO_3$ vol (ml) | Number of titrations | $V_{eq}$ (ml) | rds |
|---|---|---|---|
| 0·5 | 10 | 0·55 | 0·05 |
| 1·0 | 8 | 1·04 | 0·037 |
| 2·0 | 9 | 2·09 | 0·049 |

*Table 2. Results obtained in the determination of the $B_2O_3$ content of various frits by manual and automatic titration.*

| | $B_2O_3$ content (%) | |
|---|---|---|
| Sample | Manual titration | Automatic titration |
| FA-058A | 5·27 | 5·10 |
| RFP-1472 | 5·20 | 5·00 |
| RFP-1475 | 10·50 | 10·46 |
| ENGOBE | 9·29 | 9·03 |
| BLANCO POR | 0·62 | 0·59 |
| MIXTURE A | 14·79 | 14·42 |

*Table 3. Listing of data obtained in the titration of a frit as retrieved from a file created by the program BORIC.*

| N | V (ml) | pH | dpH/dml | N | V (ml) | pH | dpH/dml |
|---|---|---|---|---|---|---|---|
| 1 | 0·00 | 2·7 | 0·00 | 16 | 1·83 | 6·4 | 1·48 |
| 2 | 0·50 | 4·6 | 3·78 | 17 | 1·88 | 6·5 | 1·66 |
| 3 | 1·00 | 7·8 | 6·40 | 18 | 1·93 | 6·6 | 1·84 |
| 4 | 1·03 | 7·9 | 4·27 | 19 | 1·96 | 6·6 | 2·03 |
| 5 | 1·06 | 5·5 | −82.10 | 20 | 1·99 | 6·7 | 2·23 |
| 6 | 1·08 | 5·5 | 1·10 | 21 | 2·02 | 6·8 | 2·40 |
| 7 | 1·18 | 5·6 | 1·17 | 22 | 2·05 | 6·8 | 2·63 |
| 8 | 1·28 | 5·7 | 1·17 | 23 | 2·08 | 6·9 | 3·10 |
| 9 | 1·38 | 5·8 | 1·11 | 24 | 2·11 | 7·0 | 3·50 |
| 10 | 1·48 | 5·9 | 1·17 | 25 | 2·14 | 7·2 | 4·50 |
| 11 | 1·58 | 6·1 | 1·15 | 26 | 2·17 | 7·3 | 5·53 |
| 12 | 1·63 | 6·1 | 1·32 | 27 | 2·20 | 7·6 | 7·73 |
| 13 | 1·68 | 6·2 | 1·26 | 28 | 2·23 | 7·8 | 7·93 |
| 14 | 1·73 | 6·2 | 1·34 | 29 | 2·26 | 7·9 | 4·23 |
| 15 | 1·78 | 6·3 | 1·40 | | | | |

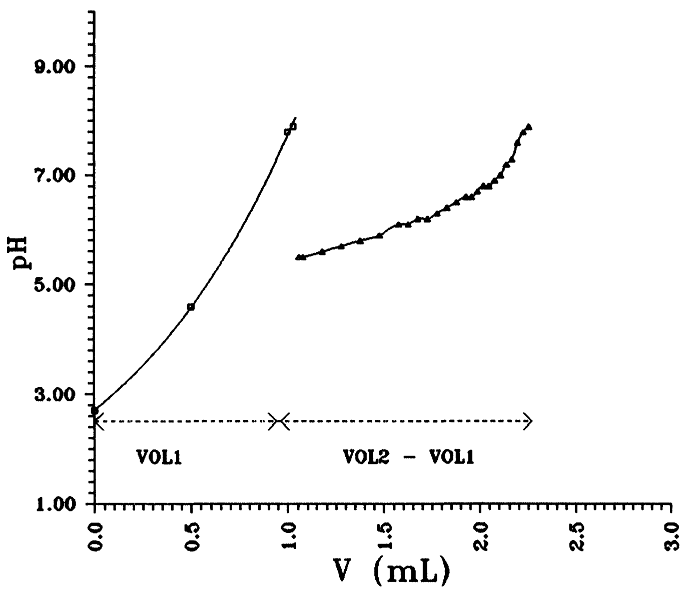

*Figure 2. Curve obtained in the titration of an aliquot of a solution resulting from the disaggregation of a frit.*

can print out tables, including ml added, pH readings and first derivative in option G, and the sample name and weight, equivalence volume and $B_2O_3$ content in option T.

Option G can be extremely useful if the end-point detection algorithm does not perform accurately – it enables manual calculation by the user.

### Results

In order to test the set-up and software, hydrochloric and boric acid, and mixtures of the two, were titrated. All possibilities, including samples with non-excess HCl, no boric acid and customary samples, were considered. In all cases, the hardware and software met expectations. Table 1 lists the results and statistical parameters obtained from data provided by BORIC. These results were obtained from the pH readings made to monitor the titration; however, the program also allows potentiometric monitoring. This avoids electrode calibration, but it somehow hinders the automatic determination of the equivalence point for boric acid because it calls for much stricter end-point calculation criteria. Therefore pH readings were used to monitor the titration according to the experimental procedure described above.

### Applications

Once the accuracy of the proposed automatic titration procedure had been checked, it was applied to the determination of $B_2O_3$ in ceramic frits. Table 2 lists the results obtained by conventional manual and automatic potentiometric titration. Table 3 and figure 2 show the listing and titration curve obtained in the titration of aliquot of the solution resulting from disaggregation of a frit.

### Conclusions

The proposed automatic potentiometric system and the program BORIC performed reliably in the titration of boric acid solutions resulting from the disaggregation of ceramic frits.

The system allows the boron in this type of product to be accurately quantified and it is a convenient, versatile and inexpensive alternative to other instrumental procedures.

The modular design of the hardware and software allows it to be readily adapted to any type of determination based on a potentiometric titration, and its automation releases the analyst from tedious routine analytical procedures and allows him or her to exert more comprehensive control over the results obtained.

### Acknowledgement

The authors are grateful to the DGCyT (the Spanish Council for Research in Science and Technology) for their financial support of Project PA 86-0033.

### References

1. Shaw, K., *Ceramic Glazes* (Elsevier, 1971).

2. Pye, P. L., Fréchette, V. D. and Kreidl, N. J., *Borate Glasses* (Plenum Press, 1978).

3. Fernández Navarro, J. M., *El Vidrio* (Consejo Superior de Investigaciones Científicas, 1985).

4. Ochandio, E., Povo, F. and Ballester, J., La fluorescencia de rayos X aplicada al análisis de fritas y esmaltes cerámicos. *Técnica Cerámica*, **174** (1986), 304–315.

5. Bennet, H. and Reed, R. A., *Chemical Methods of Silicate Analysis – A Handbook* (Academic Press, 1972).

6. Maimó, J., Far, M., Estela, J. M. and Cerdá, V., *Quim. Anal.* **5** (1986), 245–254.

7. Cerdá, V., Maimó, J., Estela, J. M., Salvá, A. and Ramis, G., *Talanta* **35** (1988), 667–669.

8. Estela, J. M., Far, M. and Cerdá, V., *Thermochimica Acta,* **153** (1989), 143–164.

9. Maimó, J., Cladera, A., Más, F., Forteza, R., Estela, J. M. and Cerdá, V., *International Journal of Environmental Analytical Chemistry,* **35** (1989), 161.

10. Cladera, A. Estela, J. M. and Cerdá V., *Talanta,* **37** (1990), 689–693.

11. Cladera, A., Estela, J. M. and Cerdá V., *Journal of Automatic Chemistry,* **12** (1990), 108.

12. Cladera, A., Estela, J. M. and Cerdá, V., *Journal of Electroanalytical Chemistry,* **288** (1990), 99–109.

